# Adolescents’ attitudes, preferences and perceived behaviours regarding healthy eating and whole grains from a gender and health interest perspective

**DOI:** 10.29219/fnr.v67.8988

**Published:** 2023-04-10

**Authors:** Anna Calvén, Karin Jonsson, Karin Wendin, Christel Larsson

**Affiliations:** 1 Department of Food and Meal Science, Kristianstad University, Kristianstad, Sweden;; 2 Division of Food and Nutrition Science, Chalmers University of Technology, Gothenburg, Sweden;; 3 Department of Food Science, University of Copenhagen, Frederiksberg C, Denmark;; 4 Department of Food and Nutrition, and Sport Science, University of Gothenburg, Gothenburg, Sweden

**Keywords:** whole grain, healthy diet, eating habits, youth, interest in health, gender

## Abstract

**Background:**

A healthy diet is important not only for the growth and development of the human body but also for the prevention of chronic diseases. However, most Swedish adolescents do not follow dietary recommendations, especially the intake of whole grain is very low. To create targeted conditions for healthy food choices amongst adolescents, comprehensive knowledge of factors related to adolescents’ unhealthy and healthy eating is needed.

**Objective:**

To investigate adolescents’ attitudes, preferences and perceived behaviours regarding healthy eating, with a specific focus on whole grains and to evaluate differences between genders and between adolescents with higher versus lower health interest.

**Design:**

A total of 1,178 Swedish adolescents responded to a questionnaire about their attitudes, preferences and perceived behabviours towards healthy eating and whole grains, and their general interest in nutrition and health. Results of girls and boys were compared, as well as adolescents with a higher versus lower health interest.

**Results:**

Displays information about healthy eating among adolescents and a comprehensive set of factors that may affect their ability to eat healthier. Overall, adolescents had a positive attitude towards food and health, but less than half felt that they managed to eat healthy. The consumption of different whole grain products was low, although the willingness to eat healthier and more whole grain was high. The most reported barrier to healthy eating, as well as increasing whole grain intake, was the unavailability of tasty healthy products, taste being even more important for adolescents with a low health interest than those with a high interest. Whole grain consumption and factors increasing the willingness of whole grain consumption were most prominent not only in adolescents with high health interest but also in girls compared with boys.

**Conclusion:**

The results show good potential to improve dietary habits amongst adolescents. Taste and availability of healthy products were rated the most important, whilst knowledge about the products’ healthiness was rated the least important, especially amongst those with low health interest. By focusing on the attitudes and preferences of adolescents, the effectiveness may increase of initiatives to improve their dietary habits.

## Popular scientific summary

- One thousand one hundred seventy-eight Swedish adolescents answered a questionnaire about healthy eating, whole grain and their perception about nutrition and health.
- Adolescents had positive attitudes towards healthy eating, but less than half felt that they managed to eat healthy.
- Taste and availability of healthy products were rated the most important for improving dietary habits, whilst knowledge about the products’ healthiness was rated the least important.
- Attitudes, preferences and perceived behaviours regarding healthy eating and whole grains may differ more between adolescents with low and high health interest, than between boys and girl.

A well-balanced diet is important for the growth and development of the human body, whilst an inadequate diet can have long-term consequences for physical and mental health (1). An adequate intake of vegetables, fruit, whole grains, fish and vegetable oils and a low intake of sugar, butter, processed/red meat, alcohol and foods with a high salt content are associated with reduced risk of diet-related diseases (1, 2). A low intake of whole grains is shown to be the primary factor contributing most to disability-adjusted life-years in Europe, according to the Institute of Health Metrics and Evaluation (3) based on data from the Global Burden of Disease. Whole grain intake has been associated with several health benefits, such as reducing the risk of all-cause mortality, heart disease, type 2 diabetes, colorectal cancer and obesity (4, 5). However, in many countries, whole grain consumption is too low compared with dietary recommendations (6–8).

In 2016–2017, the Swedish Food Agency (7) conducted a survey on dietary habits, in which 3,477 adolescents aged 12–18 participated. Compliance of dietary recommendations overall was shown to be very low, and the results revealed that only 6% had an adequate intake of whole grains, which is even lower than what was earlier found amongst adults with a corresponding share of 10% (9). This is in line with previous dietary studies that emphasize the need of efforts to increase the intake of whole grains, especially amongst children and adolescents (8). Health authorities in several countries have made efforts to motivate people to increase their intake of whole grains through various interventions and programmes (8, 10, 11). One country that has been particularly successful is Denmark with the ‘The Danish Whole Grain Partnership’ (12). A significant increase in the Danish population’s intake of whole grains was achieved by a number of efforts involving both public and industry stakeholders: whole grain labelling, campaigns targeting children, adolescents and adults, product development and partnerships (13). This demonstrates the value of joint and collaborative efforts amongst several stakeholders, at institutional, regulatory and industrial levels (8).

Recent research shows that adolescents have a relatively good understanding of healthy eating behaviours and what constitutes a healthy diet, but they struggle to apply it (14, 15). Food choice is a complex human behaviour and is influenced by several factors, such as, for example, personal liking, peers, parents, marketing and food availability (16–18). In particular, taste preferences are shown to be an important factor for long-term consumption (19–21). To successfully create targeted conditions for healthy food choices and promote healthy food habits amongst Swedish adolescents, including an increased whole grain intake, more comprehensive knowledge of factors related to adolescents’ unhealthy and healthy eating behaviours is needed. Thus, the aim of this study was to investigate adolescents’ attitudes, preferences and perceived behaviours regarding healthy eating, with a specific focus on whole grains and to evaluate possible differences between genders and between adolescents with higher versus lower health interest.

## Methods

### Project and study design

‘Help a Scientist’ is a Swedish project led by the Nobel Prize Museum that every year runs a school project with the aim of creating close cooperation amongst scientists, students and teachers. The project setup is primarily a school project, coordinated by the Nobel Prize Museum, to give high school students the possibility to learn about science and participate in the research process. The research methodology was adapted and somewhat restricted to provide the best pedagogical approach for the project and the adolescents.

The 2019 project ‘The Whole Grain Hunt’ was focused on adolescents’ attitudes, preferences and perceived behaviours regarding healthy eating, whole grains and sugar. The first part, and the focus of this study, consisted of a survey. The second part consisted of sensory tests of breads with different whole grain content and different types of sugar, accompanied by related questions (not addressed in this study). The recruitment of schools was based on advertisement in teachers’ trade journals, and recurring participation from schools that had participated in previous years’ projects and those teachers’ contacts and aimed to capture a broad geographical spread of schools, with a spread in terms of socioeconomic status. To estimate socioeconomic status, questions were posed to teachers in retrospect to provide their subjective estimation of the share of students with parents having a ‘low’ (no education *after upper secondary school*, AUSS), ‘middle’ (1–3 years of education AUSS) or ‘high’ (≥4 years of education AUSS) educational levels (estimation of the parent with the highest educational level).

### Participants

A total of 1,625 Swedish high-school students participated in this study, with the majority aged between 13 and 15 years. All 28 schools that applied in the recruitment process were included in the study and were located in different municipalities across Sweden. All the adolescents were required to have parental consent, and they were also informed by letter that participation was voluntary, and that they could withdraw from the study at any time without giving any reason. The procedure followed the Swedish Ethics Review Act, which applies to research carried out in Sweden and if the research includes processing of sensitive personal data. This study includes questions about food opinions which, according to the Data Protection Ordinance, are not classified as sensitive personal data. According to General Data Protection Regulation (GDPR), no responses to any of the questionnaires used in this study include information that can be traced to or used to identify any individual.

### Questionnaire

The questionnaire examined adolescents’ attitudes, preferences and perceived behaviours towards whole grains and high-sugar foods, as well as their general interest in nutrition and health. This study is focusing on questions about healthy eating, health interest and whole grains (Table 1). The questionnaire was in paper format, and the adolescents manually transferred the answers to an Excel file, under the teachers’ supervision. To assure data quality, random sample (10%) was carried out on how well the responses from the paper form were transformed into digital form. The percentage of accepted errors per school was set at a maximum of 5%. Schools that did not return the paper questionnaire were not included in the results since random sampling was not possible.

**Table 1:** Questionnaire with questions and answer options

1.	Background questions	○ Age ○ Gender ○ Overall, how interested are you in health?
2.	How well do the following statements about healthy eating apply to you? *(Strongly disagree; Disagree/Somewhat disagree; Neither agree nor disagree; Agree/Somewhat agree; Strongly Agree)*	○ I try to eat healthy ○ I manage to eat healthy ○ My friends usually eat healthy food ○ My family usually eats healthy food ○ When I am planning to exercise/have exercised I eat healthy
3.	How well do the following statements about unhealthy eating apply to you? *(Strongly disagree; Disagree/Somewhat disagree; Neither agree nor disagree; Agree/Somewhat agree; Strongly Agree)*	○ I get temped to buy unhealthy food in grocery stores ○ I eat more unhealthy with friends than with family
4.	What would make you eat more healthy? How well do the following statements apply to you? *(Strongly disagree; Disagree/Somewhat disagree; Neither agree nor disagree; Agree/Somewhat agree; Strongly Agree)*	○ I would eat healthier if healthy food was tastier ○ I would eat healthier if healthier food was served at home ○ I would eat healthier if school meals were tastier ○ I would eat healthier if you could buy healthy and tasty food at the school cafe ○ I would eat healthier if I had better knowledge about healthy food ○ I would eat healthier if I was more inspired
5.	Where do you get information on food and health? *(Multiple choice)*	Social media; News; Documentary; Reality; Podcast; Radio; Books; Magazines; Teachers; School nurse; Another nurse/doctor; Sports club; Parent(s); Sibling(s); Friend(s); I do not now
6.	What is your general thought about whole grains and health? *(Very healthy; Quite healthy; Neither healthy nor unhealthy; Quite unhealthy; Very unhealthy)*	
7.	What foods do you think can be whole grains? *(Multiple choice)*	Pasta; Apple; Pizza dough; Popcorn; Cereals/muesli; Milk; Nuts; Tea; Energy drink; Coffee; Meatballs; Banana; Sweet bread; Bread; Chicken; Juice; Rice; Beans; Salmon; Dried fruit; Soda; Quinoa; Bulgur; Egg; Yoghurt; Porridge; Lentils; Chocolate; Cheese; Other
8.	How often do you choose whole grain options? *(Always; Usually; Half of the times; Sometimes; Never)* *(Do not eat it)*	○ Bread ○ Pasta ○ Rice ○ Breakfast cereals ○ Porridge
9. How often do you talk about whole grain in your family? *(Very often; Quite often; Sometimes; Rarely; Never)*	
10.	If you knew that it was healthier to choose whole grain foods, how well do the following statements apply to you? *(Strongly disagree; Disagree/Somewhat disagree; Neither agree nor disagree; Agree/Somewhat agree; Strongly Agree)*	○ I would eat more whole grains if it was served more at school ○ I would eat more whole grains if it was served more at home ○ I would eat more whole grains if more options were available in grocery stores ○ I would eat more whole grains if more options were available in the cafe ○ I would eat more whole grains if the products tasted better ○ I would eat more whole grains if the products looked tastier ○ Products with whole grains should taste like whole grains ○ If I know that the product contains whole grains, it appeals to me ○ Don’t want to eat more whole grain, already eat a lot ○ Don’t want to eat more whole grain, don’t care about healthy food
11.	How well do the following statements apply to you when considering choosing more of following whole grain products? *(Strongly disagree; Disagree/Somewhat disagree; Neither agree nor disagree; Agree/Somewhat agree; Strongly Agree)* *(Already eat a lot of it; Do not eat it)*	○ More whole grain bread ○ More whole grain pasta ○ More whole grain rice ○ More whole grain porridge ○ More fast food with whole grain ○ More whole grain cereals/muesli

### Data analysis

**Fig. 1:**
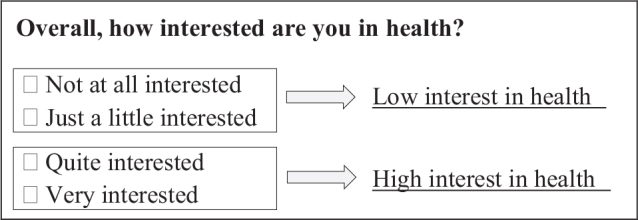
Two groups, low versus high interest in health, were created based on an initial question about the level of interest in health. ‘Not at all interested’ and ‘just a little interested’ = Low interest. ‘Quite interested’ and ‘Very interested’ = High interested in health.

Responses were converted to numeric values. Descriptive analysis was used to analyse collected data for frequency, percentage and mean. Girls and boys were compared. In addition, two groups were created based on an initial question about the level of interest in health (from high interest to no interest) (Fig. 1). The answers from the questions were converted to a five-graded scale format, from ‘lowest’ to ‘highest’ (e.g. Strongly disagree, Disagree/Somewhat disagree, Neither disagree nor agree, Agree/Somewhat agree, Strongly agree), with the underlying assumption of equal steps between the choices, equalling 1, 2, 3, 4 and 5.

Student’s *t*-test was performed to evaluate significant differences (*P* < 0.05) between girls and boys, as well as between the adolescents with low versus high interest in health. Statistical analyses were conducted with Excel 365 and Jamovi 2.2.5. The significance level of 0.05 was retained for the large number of tests, hence, increasing the risk of obtaining significances by chance, to allow for exploring a more distinct pattern of potential differences between genders and health interest.

## Results

This study displays information about adolescents and a comprehensive set of factors that may affect their ability to eat healthier. A total of 1,178 Swedish adolescents from 21 schools were included in the results: girls 584 (49.6%); boys 578 (49.1%); non-binary 13 (1.1%); gender unknown/missing information 3 (0.2%). The schools included represented a broad geographical spread (from Malmö and Kalmar in the South to Åre and Kiruna further North) from municipalities of different sizes (from Torsås and Tranemo of a few thousand inhabitants to Stockholm and Gothenburg with several hundred thousand). The recruitment process aimed at targeting schools with students from families of different socioeconomic status. To support the spread of socioeconomic status, teachers were asked in retrospect to subjectively estimate educational level of the parents. This was not perceived as a straightforward task; hence, information for eight of 21 schools was obtained. The result of the eight schools show a spread (3 ‘high’, 2 ‘middle’ and 3 ‘low’). Of the participants (*n* = 1,178), 458 reported a lower interest in health (LIH), whilst 712 reported a higher interest in health (HIH), Table 2. A total of eight respondents did not answer the question about level of interest in health and are considered as missing data. Please note that missing data are not included in the results illustrated in figures and tables.

**Table 2:** Distribution of answers to the question about level of interest in health (missing data *n* = 8, not included in the results)

Overall, how interested are you in health? *N* (%)		
Not at all interested	69 (5.9)	Lower interest: 458 (39.1)
Just a little interested	389 (33.2)	
Quite interested	494 (42.2)	Higher interest: 712 (60.9)
Very interested	218 (18.6)	

**Table 3:** Gender distribution of the low (LIH) versus high interest in health (HIH) groups

*N* (%)	Girls	Boys	Non-Binary
Lower interest in health	222 (48.5)	231 (50.4)	5 (1.1)
Higher interest in health	359 (50.6)	343 (48.3)	8 (1.1)

Missing data, not included in the results, are distributed as follows: girls *n* = 3, boys *n* = 4. In addition, three respondents had not answered the question about gender, which means that the total number of missing data for this table is *n* = 10.

### Healthy eating

**Fig. 2:**
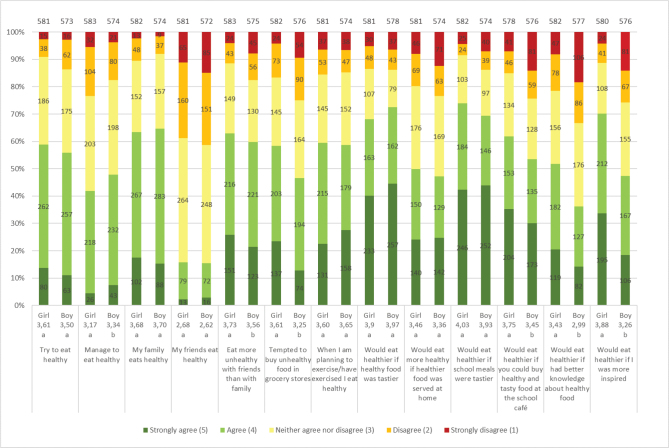
Results for the question: ‘How well do the following statements about healthy and unhealthy eating apply to you?’. The numbers in the bars show the distribution of the number of responses for each gender, and its percentage equivalent is indicated on the diagram axis. The mean values (1–5) for each question are shown below each graph bar, beneath girls and boys. Significant differences between girls and boys are illustrated by the letters a and b. The total number of girls and boys included in the results of the study were 584 and 578, respectively.

Adolescents’ attitudes, preferences and perceived behaviours regarding healthy eating are shown in Fig. 2, comparing girls and boys and comparing adolescents with low versus high interest in health. Of the girls and boys, 59% and 56%, respectively, tried to eat healthy, but fewer felt they succeeded (girls 42%; boys 48%). For both genders, taste (girls 68%; boys 72%) was important for a healthier diet, but for girls, inspiration (70%) was also a valuable factor. Several adolescents reported that their family ate healthy (girls 63%; boys 65%), whilst fewer reported that their friends ate healthy (girls 16%; boys 15%). Unhealthier food was eaten together with friends than with family (girls 63%; boys 60%). Significant, although relatively small, differences were found between the genders, where a larger number of boys reported they managed to eat healthy, whilst a larger number of girls reported they ate more unhealthy with friends than family and were tempted to buy unhealthy foods in grocery stores, and they would eat healthier if they could buy healthy and tasty foods at the school café, had better knowledge about healthy food and were more inspired (Fig. 2). The main sources from which the adolescents received information about food and health were social media (girls 74%; boys 65%), parents (girls 67%; boys 59%) and teachers (girls 56%; boys 54%).

**Fig. 3:**
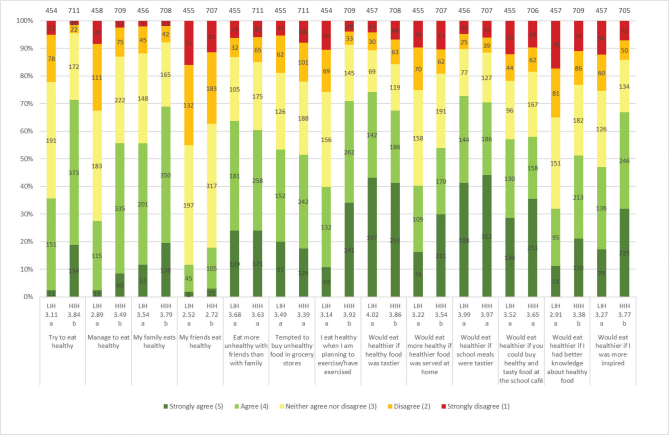
Results for the question: ‘How well do the following statements about healthy and unhealthy eating apply to you?’. The numbers in the bars show the distribution of the number of responses for each LIH and HIH group, and its percentage equivalent is indicated on the diagram axis. The mean values (1–5) for each question are shown below each graph bar, beneath LIH and HIH. Significant differences between LIH and HIH are illustrated by the letters a and b. The total number of LIH and HIH respondents included in the results of the study were 458 and 712, respectively.

Division into groups based on high (HIH) and low (LIH) health interest resulted in more significant differences in the answers than between groups based on gender (Fig. 3). A higher number of the HIH group tried to eat healthy (71%) and considered themselves successful in doing so (56%), especially when they had exercised or were planning to exercise (71%), compared to the LIH group. For both HIH and LIH, tasty products were important for a healthier diet, and more so for LIH (74 and 68%, respectively). This was also reflected in a general apprehension of tastier food in the schools to eat healthier, although no significant difference was observed between the groups. A higher number of the HIH group reported that the family eats healthy (69%), and inspiration was also a valuable factor, foremost for HIH (67%). More knowledge appeared to be the least important factor for motivating the adolescents to eat healthier, and even less so for the LIH group for which only 32% considered it helpful (51% for HIH). The main sources from which the two groups received information about food and health were social media (LIH 62%; HIH 72%), parents (LIH 53%; HIH 69%) and teachers (LIH 53%; HIH 57%).

### Whole grain products

**Fig. 4:**
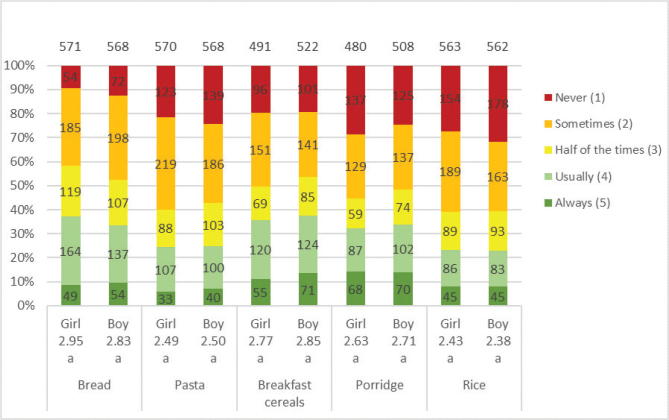
Results for the question: ‘For the following foods, how often do you choose whole grain options?’. The numbers in the bars show the distribution of the number of responses for each gender, and its percentage equivalent is indicated on the diagram axis. The mean values (1–5) for each question are shown below each graph bar, beneath girls and boys. Significant differences between girls and boys are illustrated by the letters a and b. For this question, the adolescents also had the option to answer ‘Do not eat it’. The number of participants answered that they ‘do not eat it’ were distributed as follows: bread (girl 17; boy 5), pasta (girl 12; boy 6), breakfast cereals (girl 92; boy 48), porridge (girl 106; boy 68) and rice (girl 19; boy 11). The total number of girls and boys included in the results of the study was 584 and 578, respectively.

**Fig. 5:**
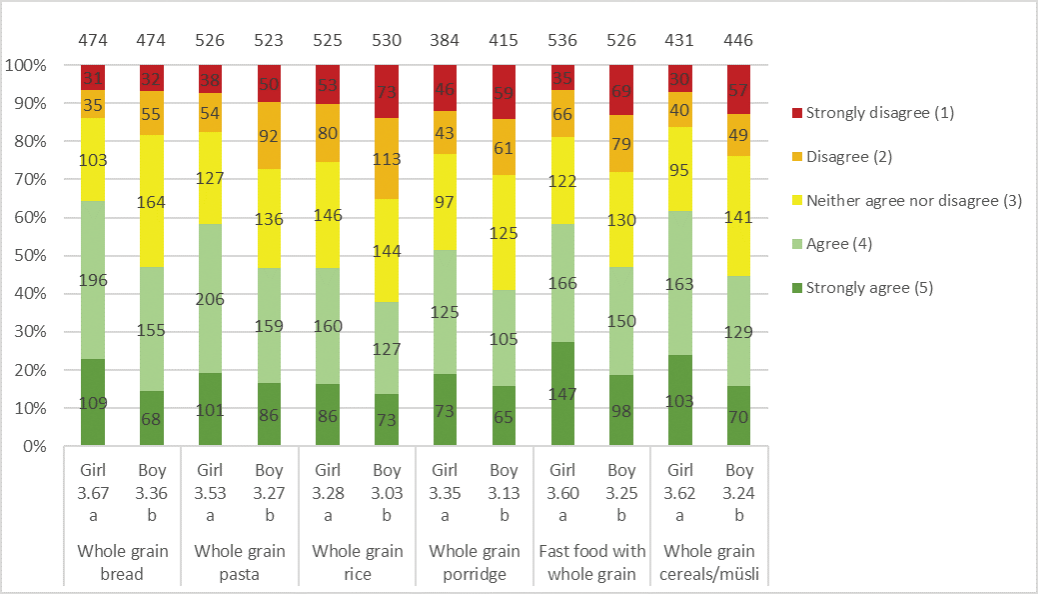
Results for the question: ‘How well do the following statements apply to you when considering choosing more of following whole grain products’. The numbers in the bars show the distribution of the number of responses for each gender, and its percentage equivalent is indicated on the diagram axis. The mean values (1–5) for each question are shown below each graph bar, beneath girls and boys. Significant differences between girls and boys are illustrated by the letters a and b. For this question, the adolescents also had the option to answer: ‘Already eat a lot of it’ and ‘Do not eat it’ (Table 5). The total number of girls and boys included in the results of the study was 584 and 578, respectively.

**Fig. 6:**
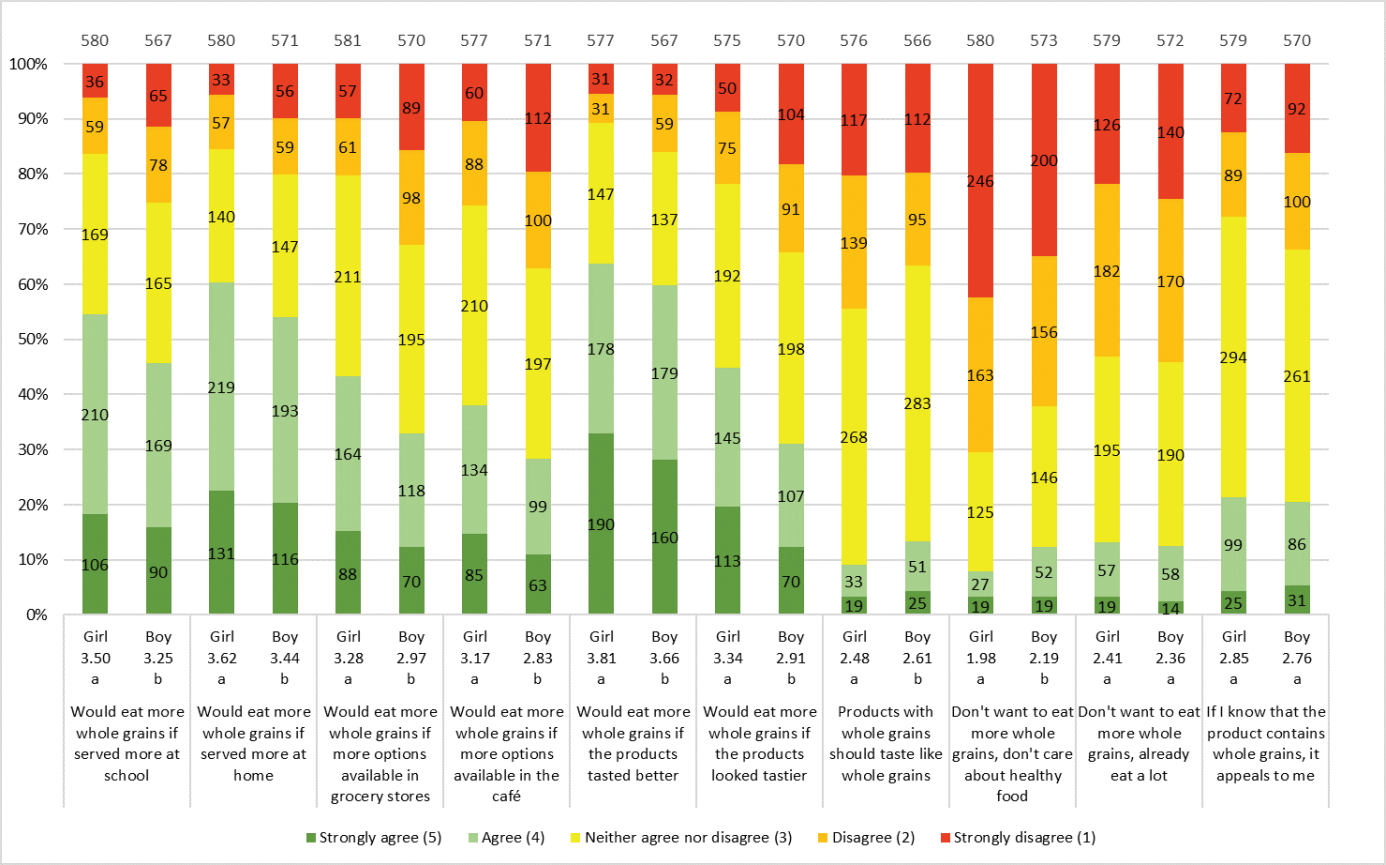
Results for the question: ‘If you knew that it was healthier to choose whole grain foods, how well do the following statements apply to you?’. The numbers in the bars show the distribution of the number of responses for each gender, and its percentage equivalent is indicated on the diagram axis. The mean values (1–5) for each question are shown below each graph bar, beneath girls and boys. Significant differences between girls and boys are illustrated by the letters a and b. The total number of girls and boys included in the results of the study was 584 and 578, respectively.

The majority of adolescents considered whole grains to be quite healthy or very healthy (girls 87%; boys 79%). Both genders were familiar with whole grain products when it comes to bread, pasta and cereals but were less aware of foods such as sweet bread and popcorn (Table 4). Overall, girls had better knowledge than boys. The most chosen whole grain products for both genders were bread and breakfast cereals, although the percentage of girls and boys choosing these products ‘usually’ or ‘always’ was ≤37% (Fig. 4). However, many of the adolescents were willing to eat additional products containing whole grains; girls were significantly more than boys; bread, muesli, pasta and fast food are those of highest interest (Fig. 5). The factors most influencing the willingness to choose more whole grain products were whether whole grain products tasted better (girls 64%; boys 60%) and whether whole grain products were served more often at home (girls 60%; boys 54%) or at school (girls 54%; boys 46%) (Fig. 6). Few adolescents wanted whole grain products to taste like whole grain (girls 9%; boys 13%), and a moderate number considered it important for increased consumption to know the products contain whole grain (girls 21%; boys 20%). Girls were significantly more willing about eating additional whole grain products overall, with the most prominent difference coming from whether whole grain products looked tastier.

**Table 4:** What foods do you think can be whole grains? (Multiple choice)

% (*n*)	Girl n = 584	Boy n = 577	LIH n = 457	HIH n = 712
Bread	84.6 (494)	72.4 (418)	72.9 (333)	82.9 (590)
Pasta	76.2 (445)	65.9 (380)	66.5 (304)	74.3 (529)
Cereals/muesli	73.8 (431)	63.6 (367)	61.5 (281)	73.7 (525)
Porridge	53.3 (311)	43.2 (249)	44.6 (204)	51.1 (364)
Bulgur	49.5 (289)	36.2 (209)	40.3 (184)	45.1 (321)
Rice	47.9 (280)	40.0 (231)	41.6 (190)	45.8 (326)
Pizza dough	44.0 (257)	41.9 (242)	39.2 (179)	45.9 (327)
Sweet bread	38.4 (224)	38.3 (221)	36.3 (166)	40.0 (285)
Quinoa	31.8 (186)	18.4 (106)	24.1 (110)	26.4 (188)
Lentils	24.8 (145)	21.3 (123)	23.4 (107)	23.2 (165)
Beans	19.7 (115)	20.8 (120)	21.4 (98)	19.5 (139)
Nuts	13.0 (76)	15.9 (92)	14.0 (64)	15.2 (108)
Popcorn	8.4 (49)	9.7 (56)	7.0 (32)	10.5 (75)
Milk	7.5 (44)	8.1 (47)	8.3 (38)	7.4 (53)
Egg	5.3 (31)	7.3 (42)	7.7 (35)	5.5 (39)
Salmon	4.8 (28)	6.8 (39)	7.2 (33)	5.2 (37)
Apple	4.6 (27)	5.2 (30)	6.1 (28)	4.1 (29)
Chicken	4.6 (27)	4.7 (27)	5.0 (23)	4.6 (33)
Banana	4.5 (26)	4.7 (27)	5.0 (23)	4.5 (32)
Meatballs	4.3 (25)	4.3 (25)	5.0 (23)	4.1 (29)
Dried fruit	4.1 (24)	3.5 (20)	4.6 (21)	3.7 (26)
Yoghurt	3.6 (21)	3.8 (22)	3.9 (18)	3.7 (26)
Chocolate	3.3 (19)	4.5 (26)	4.6 (21)	3.5 (25)
Cheese	3.3 (19)	4.0 (23)	4.6 (21)	3.2 (23)
Coffee	3.1 (18)	4.7 (27)	4.8 (22)	3.7 (26)
Juice	2 .4(14)	2.9 (17)	2.6 (12)	2.7 (19)
Tea	2.1 (12)	2.6 (15)	2.6 (12)	2.4 (17)
Soda	2.1 (12)	2.3 (13)	3.1 (14)	1.7 (12)
Energy drink	1.4 (8)	2.4 (14)	1.8 (8)	2.4 (17)

Numbers and percentages are reported for boys, girls and groups with low versus high interest in health. Foods written in italics can be whole grains, in terms of content of cereal based ingredients.

**Fig. 7:**
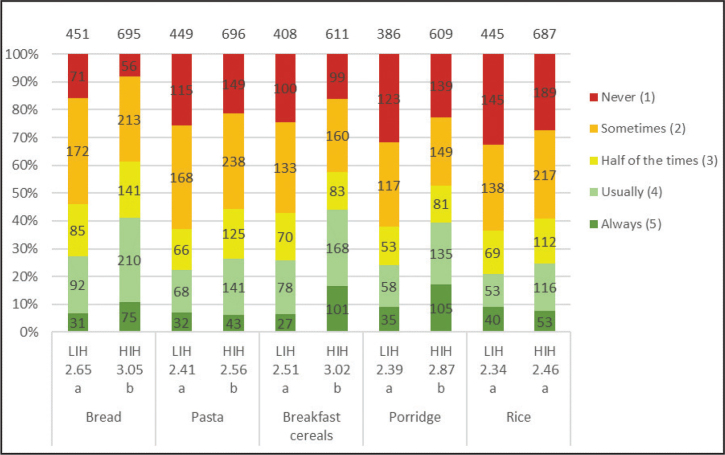
Results for the question: ‘For the following foods, how often do you choose whole grain options?’. The numbers in the bars show the distribution of the number of responses for each LIH and HIH group, and its percentage equivalent is indicated on the diagram axis. The mean values (1–5) for each question are shown below each graph bar, beneath LIH and HIH. Significant differences between LIH and HIH are illustrated by the letters a and b.

**Fig. 8:**
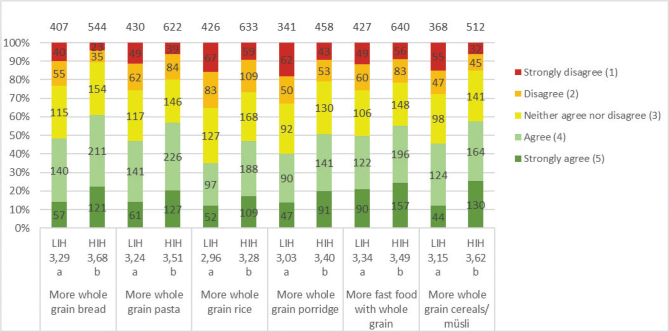
Results for the question: ‘How well do the following statements apply to you when considering choosing more of following whole grain products?’. The numbers in the bars show the distribution of the number of responses for each LIH and HIH group, and its percentage equivalent is indicated on the diagram axis. The mean values (1–5) for each question are shown below each graph bar, beneath LIH and HIH. Significant differences between LIH and HIH are illustrated by the letters a and b. For this question, the adolescents also had the option to answer: ‘Already eat a lot of it’ and ‘Do not eat it’ (Table 5). The total number of LIH and HIH respondents included in the results of the study was 458 and 712, respectively.

**Fig. 9:**
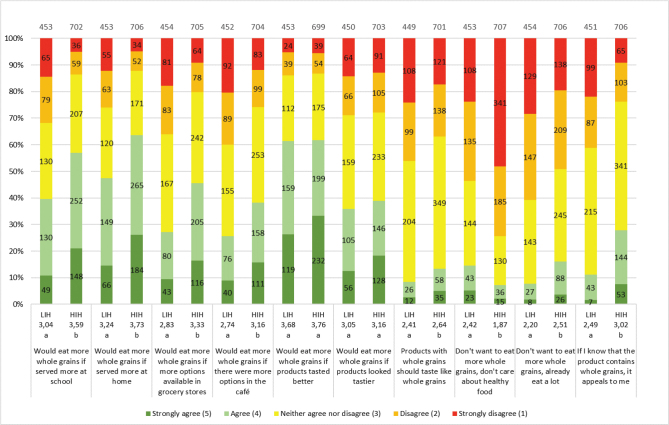
Results for the question: ‘If you knew that it was healthier to choose whole grain foods, how well do the following statements apply to you?’. The numbers in the bars show the distribution of the number of responses for each LIH and HIH group, and its percentage equivalent is indicated on the diagram axis. The mean values (1–5) for each question are shown below each graph bar, beneath LIH and HIH. Significant differences between LIH and HIH are illustrated by the letters a and b. The total number of LIH and HIH respondents included in the results of the study was 458 and 712, respectively.

In the HIH and LIH groups, 86 and 77%, respectively, of the adolescents considered whole grains to be quite healthy or very healthy. Both groups were familiar with whole grain products, especially bread, pasta and cereals, but were less aware of foods such as sweet bread and popcorn (Table 4). Overall, the HIH group had better knowledge of foods that could be made of whole grain than the LIH group. The most commonly chosen whole grain products for both groups were bread (HIH 41%; LIH 27%) and breakfast cereals (HIH 44%; LIH 26%) (Fig. 7). The HIH group, however, chose whole grain products significantly more often than the LIH group, except for rice where no difference was found. Both groups were willing to eat additional products containing whole grains, and the HIH group is significantly more than LIH of all products (Fig. 8). For all factors influencing the willingness to choose more whole grain products, a significantly larger number in the HIH group were willing to eat more, except for if whole grain products tasted better or looked tastier, where no significant differences were seen (Fig. 9). The factor of whole grain being served more at home had highest number of willingness to eat more whole grain for the HIH group (64%), although if whole grain products tasted better had highest number overall, considering both groups (HIH 62%; LIH 61%). Few of the adolescents considered the specific whole grain taste (HIH 13%; LIH 8%), or information about whole grain content (HIH 28%; LIH 11%), to be important for eating more whole grain.

**Table 5:** The number of adolecents responing that they ‘Already eat a lot of it’ or ‘Do not eat it’ to the question: ‘How well do the following statements apply to you when considering choosing more of following whole grain products’ (Figs. 5 and 8)

N	Whole grain bread		Whole grain pasta		Whole grain rice		Whole grain porridge		Fast food with whole grain		Whole grain cereals/müsli	
	Girl	Boy	Girl	Boy	Girl	Boy	Girl	Boy	Girl	Boy	Girl	Boy
Already eat a lot of it	91	87	46	46	37	34	77	78	19	31	75	85
Do not eat it	18	11	15	7	22	10	120	83	27	16	76	44
	LIH	HIH	LIH	HIH	LIH	HIH	LIH	HIH	LIH	HIH	LIH	HIH
Already eat a lot of it	37	143	20	75	19	54	31	128	15	34	38	125
Do not eat it	12	19	9	13	12	20	83	123	12	32	48	73

For this question, the adolescents also had the option to answer (‘do not eat it’), referring to the product in general. The number of participants answered that they ‘do not eat it’ were distributed as follows: bread (LIH 7; HIH 16), pasta (LIH 6; HIH 13), breakfast cereals (LIH 50; HIH 91), porridge (LIH 74; HIH 100) and rice (LIH 10; HIH 20). The total number of LIH and HIH respondents included in the results of the study was 458 and 712, respectively.

## Discussion

### Healthy eating

The present study shows that many adolescents are interested in health and try to eat healthy, but less than half feel that they succeed. According to Calvert et al. (14), secondary school students have a good understanding of healthy food choices, but they do seldom practise them. The need for improvement in eating habits shown in this study confirms previous studies that reported poor compliance with general dietary recommendations by youths (7).

The majority of adolescents responded that they would eat healthier if healthy food was tastier, as well as if school meals were tastier, which was considered the most important feature to increase the willingness to consume healthier. The findings are in line with previous studies showing the importance of sensory properties and taste to influencing food choice (18, 22). Furthermore, healthy foods being served at home and in public meals, such as schools, were considered to be of higher importance than factors such as increased knowledge. In fact, we found that knowledge about health was considered to be the least important feature for stimulating healthier food choices. This shows the importance of complementary strategies in addition to education and information directly to the end consumer, directed to, for example, companies and public institutions for increased innovation and availability. The findings of current whole grain consumption and factors increasing the willingness of whole grain consumption were, not surprisingly, most prominent not only in adolescents with high health interest but also in girls compared with boys. The correlation between health interest and gender and the current dietary habits and willingness for healthier habits may suggest an increased efficiency of public health interventions by tailoring the strategies based on health interest and gender.

However, turning the interpretation of the results around, encouraging young people’s interest in health, might be an additional way to improve dietary habits, since adolescents with higher health interest tried to eat healthy, and also stated that they succeeded in eating healthy to a greater extent, than those with a low health interest. Adolescents with a higher level of exercise reported to eat healthier to a higher extent than those with a lower level of exercise, which possibly may further underline the importance of inspiring adolescents’ interest in health-promoting activities as a means to improve their dietary habits.

Social media was mentioned as a valuable source of information about food and health, especially by girls. According to a recent study, the frequency of food products seen in social media advertising is positively related to the willingness to consume these products (17). Social media are proposed to be used for marketing strategies promoting healthy eating habits amongst children and adolescents (17, 23), as well as protecting youth from marketing of unhealthy foods by implementing legislation and policies that prevent this exposure (24, 25). However, further research is needed to investigate how social media advertising influences young people’s food choices and their actual consumption behaviour (25). In addition, more than half of the participants responded that parents and teachers were an important source of information about food and health, which indicates that adults still have a role to play in teaching, motivating, inspiring and supporting young people to healthy choices.

Given that the majority of participants ate unhealthier with friends than with family, the access to nutritious and tasty options, also outside schools and homes, can still be of importance, which is in line with previous studies showing that low availability of nutritious food is perceived as a barrier to healthy food choices (14, 18).

### Whole grain products

Bread and breakfast cereals were reported to be the whole grain products most chosen by the Swedish adolescents, which is in line with the result of the study by Kamar (18), where factors influencing UK adolescents’ whole grain intake were investigated. In the present study, perceptions, attitudes and preferences were investigated with consideration of gender and health interest. The adolescents with a high interest in health compared to those with less health interest, and girls compared to boys, tended to choose whole grain products significantly more often. These results are in accordance with the study of Pohjanheimo et al. (26), investigating Finnish adolescents’ attitudes towards health and whole grain. However, the overall low share of participants who always or usually responded that they chose whole grain products emphasize the need of improvement in whole grain intake. Interventions aiming at improving the whole grain intake in adolescents may have a good chance to succeed, since the willingness to consume whole grain products was surprisingly high – quite strikingly high if also including those that were neutral, covering up to almost 90% of the adolescents, depending on gender or health interest, and never lower than well over 60%, regardless of grouping.

In line with earlier studies (19–21), the most important factor influencing adolescents’ willingness to eat more whole grains was if whole grain products tasted better. The lack of availability was also found to be a contributing factor influencing the willingness to eat more whole grains, both at home and at school. Overall, the main barrier to increased consumption seems to be the limited availability of healthy options that are tasty, in line with the results of Pohjanheimo et al. (26), where taste and freshness were important bread choice motives, but dependent on what is available at home. Availability at home as well as in school was found also in the present study to important factors for healthy eating and consuming more whole grain products. The results by Kamar et al. (18) corroborate our findings that there is a need to tailor products for teenagers and to increase the availability and variety of whole grain products, as well as improving sensory appeal. This is also suggested by (27) who found that sensory attractiveness, home availability and self-efficacy were related to increased whole grains consumption. To understand what features of a bread that adolescents find attractive, we let the same subjects (*n* = 931) in a parallel study ranking important features for a tasty soft bread (most important to least important), according to their perceptions and desires when asked to rank a number of written alternatives (i.e. not by tasting): 1) softness 68%; 2) juicy 53%; 3) fluffy 48%; 4) fibre 47%; 5) homemade 44%; 6) organic 44%; 7) whole grain 39% (unpublished data). In the same parallel study, sensory tests were made of breads with as similar features as possible, including sugar content, but with different whole grain content. We could demonstrate that the acceptability of bread with 0 and 32% whole grain (dry weight) did not differ, and that the acceptability of those breads was almost equal to bread containing 51% whole grain content – in unprepared state (not toasted, neither spreads nor cold cuts, which may increase acceptability). We suggest that these results may well be extrapolated to the general population of all ages, since the older the population is, the healthier the food choices are preferred, according to surveys of Swedish adolescents and adults, respectively, by the Swedish Agency (7, 9).

A majority of the adolescents in the present study were aware that whole grain is healthy and were familiar with common whole grain product categories (bread, pasta and cereals/muesli). However, it should be noted that the results are likely to have been biased as the adolescents knew that they were participating in a research project called ‘the whole grain hunt’. Attempting to avoid this type of bias, the teachers got careful instructions of not talking about wholegrain and health before the adolescents had answered all the questions in the survey. Considering the already high knowledge of whole grain products being healthy, this leaves less room for communication and education strategies around whole grain and health, although the bias needs to be kept in mind. However, accordingly, increased knowledge about whole grain and wholegrain products – and also sensing the taste of whole grains – was less important factor, according to the answers of most of the adolescents regarding their willingness to increase their whole grain intake.

Findings of our study show that several factors influence food choices and whole grain consumption, such as taste, availability, family and friends, which underline that food choice is a complex human behaviour (16, 18). Hence, a variety of efforts are likely to be required to promote healthy eating habits (8, 10, 11), of which taste and availability were the most important factors, according to our study. The successful outcome of the ‘Danish Whole Grain Partnership’ (19) corroborates the importance of increased availability of whole grain products, with an increase of whole grain products on the Danish market of 577% (190 to 1,097 products) in barely 10 years, after the implementation of a broad partnership (12). Even if knowledge was found to be the least important factor for increasing adolescents whole grain intake in our study, targeted communication campaigns and the creation of a whole grain logotype were part of the strategy of the partnership, in addition to increased innovation and availability of whole grain products. Potentially, the Danish strategy can be transferred to other countries (28). It is important to consider, however, that there are differences between countries when it comes to food culture, even within the Nordic countries, and the strategies may need to be tailored to suit different societies (6, 29, 30).

As a result of the project setup of this study, primarily being a school project adapted to provide a pedagogical approach for the adolescents to participate and contribute to scientific work, the research methodology was impacted. A major limitation was the non-digital paper format of the survey, due to the project’s wish to increase the understanding and involvement of the students in the research process and processing of the data. However, from a scientifical point of view, the data collection was negatively impacted as a result of the students manually transferring raw data from the survey in paper format to Excel. To verify correct data entry, all Excel sheets were reviewed at a superficial level (having a reasonable answer in every cell), and a detailed sampling was done, carefully reviewing 10% of the data transfer between the paper survey and the Excel sheet for each school, removing those schools with more than 5% mistakes which resulted in a loss of 30% of the answers. Through this effort, the exclusion rate became high, but the correctness of the data entry of the remaining answers was increased. The remaining number of over a thousand participating adolescents was large, compared with the similar studies by Kamar et al. (18) and Pohjanheimo et al. (26), including 50 and 61–104 participants, respectively. Moreover, the geographical coverage was good, with schools from small and large municipalities and students with a close to identical share of both genders. The socioeconomical status of the participants and their families was difficult to assess, but indicated a spread, measured by the teachers’ estimation of the parents’ educational level. The questionnaire was large and comprehensive, and participation of such extensive survey was enabled, thanks to teachers providing and supervising the answering as part of the regular schoolwork. However, due to the extensiveness of the survey, the answers may be less considered towards the end.

In this study, attitudes, preferences and perceived behaviours of adolescents have been evaluated, and not a real-life setting, including actual food choices in stores/schools or actual intakes. However, the findings of this study provide information about the perceived needs of adolescents to improve their diets, which, in turn, may increase the effectiveness of ongoing interventions and actions, and those in plan, and further studies in real-life settings.

## Conclusion

This study investigated adolescents’ attitudes, preferences and perceived behaviours regarding healthy eating and whole grains from a gender and health interest perspective. Overall, adolescents had a positive attitude towards healthy eating, but less than half felt that they managed to eat healthy. Reported consumption of different whole grain products was low, although the willingness to eat healthier and more whole grain was surprisingly high, showing large potential for targeted public health interventions. The taste and availability of healthy products were reported to be the most important factors to increase the willingness of eating healthier and consuming more whole grain products, whilst increased knowledge of healthier foods was considered the least important factor. On the other hand, girls (that also reported a higher health interest) and adolescents of both gender with higher health interest, respectively, tended to report a perceived healthier current diet, which indicates that learning about food and health may stimulate healthier eating to a certain extent. Our results may help increasing the efficacy of dietary interventions, providing guidance around the attitudes, preferences and perceived behaviours of adolescents, where the availability of tasty healthy products seems to be more important than knowledge about the healthiness of food products. Moreover, attitudes, preferences and perceived behaviours around healthy foods seem to differ more between adolescents with low versus high health interest, than between boys versus girls. The findings of this study may provide useful information and serve as inspiration for product development, public health interventions and further research on healthy eating and whole grain consumption.

## Supplementary Material

Adolescents’ attitudes, preferences and perceived behaviours regarding healthy eating and whole grains from a gender and health interest perspective
