# Supplementary material for: Adolescents’ attitudes, preferences and perceived behaviours regarding healthy eating and whole grains from a gender and health interest perspective
Source: Food Nutr Res. 2023 Apr 10;67:10.29219/fnr.v67.8988. doi: 10.29219/fnr.v67.8988 (PMC10158873; doi:10.29219/fnr.v67.8988)
Supplement: Adolescents’ attitudes, preferences and perceived behaviours regarding healthy eating and whole grains from a gender and health interest perspective [file FNR-67-8988-s1.pdf]

Utvärdering av attityd kring mat, hälsa och kolhydrater

Detta är en enkät med frågor om mat, hälsa och kolhydrater. Inga namn samlas in och resultaten kommer att redovisas på gruppnivå, vilket innebär att ingen då kommer att se hur just du har svarat. Istället för namn får du ange en kod som du får av din lärare. Den är till för att vi ska kunna koppla ihop dina enkäter med varandra utan att veta vem du är. Studien är frivillig och du kan när som helst, utan att ange orsak, avbryta din medverkan.

Tack för din hjälp!
Forskarhjälpen
och forskarna
Karin Jonsson, Chalmers tekniska högskola
Christel Larsson, Göteborgs universitet

Vänligen skriv din personliga kod:_________

Nedan ställs 6 bakgrundsfrågor följt av 18 frågor om mat och hälsa (totalt 24).
Välj det svarsalternativ som passar bäst in på dig. Välj endast ett svar, även om det inte stämmer exakt.

1. **Vilken årskurs går du i?**
   - ☐ 7: an
   - ☐ 8: an
   - ☐ 9: an

2. **Hur gammal är du?**
   - ☐ 12 år
   - ☐ 13 år
   - ☐ 14 år
   - ☐ 15 år
   - ☐ 16 år

   Annan ålder:_______

3. **Vilket kön har du?**
   - ☐ Tjej
   - ☐ Kille
   - ☐ Annat

4. Hur ofta tränar du? (Räkna <u>inte</u> med idrotten i skolan.)

☐ Aldrig eller nästan aldrig

☐ 1-3 gånger i månaden

☐ 1-3 gånger i veckan

☐ 4 eller fler gånger i veckan

5. Hur mycket tittar/spelar du i genomsnitt på mobil/surfplatta/dator/TV, utöver skoltid?

☐ Mindre än 1 timme per dag

☐ 1-2 timmar per dag

☐ 3-4 timmar per dag

☐ 5 timmar eller mer per dag

6. Hur intresserad är du av hälsa generellt?

☐ Inte intresserad alls

☐ Lite intresserad

☐ Ganska intresserad

☐ Mycket intresserad

Nedan kommer vi att ställa frågor kring hur du tänker kring mat och hälsa. Välj endast <u>ett</u> svar. Hittar du inte ett svar som stämmer exakt med din uppfattning, välj det alternativ som ligger närmast. För vissa frågor kan fler svar väljas, då har vi skrivit ut det. Annars gäller <u>ett</u> svar. *Kom ihåg! Det finns inga "rätt" eller "fel" svar. Det är bara din egen åsikt som är intressant!*

7. Hur väl stämmer följande alternativ när det kommer till att äta hälsosamt/nyttigt?

Jag försöker att äta nyttigt

☐ Stämmer mycket dåligt

☐ Stämmer ganska dåligt

☐ Stämmer varken bra eller dåligt

☐ Stämmer ganska bra

☐ Stämmer mycket bra

Jag tycker att jag lyckas bra med att äta nyttigt

☐ Stämmer mycket dåligt

☐ Stämmer ganska dåligt

☐ Stämmer varken bra eller dåligt

☐ Stämmer ganska bra

☐ Stämmer mycket bra

Jag känner mig säker på vad som är nyttigt att äta

☐ Stämmer mycket dåligt

☐ Stämmer ganska dåligt

☐ Stämmer varken bra eller dåligt

☐ Stämmer ganska bra

☐ Stämmer mycket bra

Det är förvirrande att olika personer säger att olika saker är nyttigt att äta

☐ Stämmer mycket dåligt

☐ Stämmer ganska dåligt

☐ Stämmer varken bra eller dåligt

☐ Stämmer ganska bra

☐ Stämmer mycket bra

Mina kompisar äter oftast nyttigt

☐ Stämmer mycket dåligt

☐ Stämmer ganska dåligt

☐ Stämmer varken bra eller dåligt

☐ Stämmer ganska bra

☐ Stämmer mycket bra

Min familj äter oftast nyttigt

☐ Stämmer mycket dåligt

☐ Stämmer ganska dåligt

☐ Stämmer varken bra eller dåligt

☐ Stämmer ganska bra

☐ Stämmer mycket bra

Jag tror att skolan oftast serverar nyttig mat

☐ Stämmer mycket dåligt

☐ Stämmer ganska dåligt

☐ Stämmer varken bra eller dåligt

☐ Stämmer ganska bra

☐ Stämmer mycket bra

Det finns nyttiga mellanmål i närheten att handla på raster

☐ Stämmer mycket dåligt

☐ Stämmer ganska dåligt

☐ Stämmer varken bra eller dåligt

☐ Stämmer ganska bra

☐ Stämmer mycket bra

Jag äter ofta nyttigt när jag ska träna eller har tränat

☐ Stämmer mycket dåligt

☐ Stämmer ganska dåligt

☐ Stämmer varken bra eller dåligt

☐ Stämmer ganska bra

☐ Stämmer mycket bra

Jag blir dålig i magen av nyttig mat

☐ Stämmer mycket dåligt

☐ Stämmer ganska dåligt

☐ Stämmer varken bra eller dåligt

☐ Stämmer ganska bra

☐ Stämmer mycket bra

8. Hur väl stämmer följande alternativ in när det kommer till att *inte* äta nyttigt - alltså att äta onyttigt?

Stress får mig att äta onyttigt

☐ Stämmer mycket dåligt

☐ Stämmer ganska dåligt

☐ Stämmer varken bra eller dåligt

☐ Stämmer ganska bra

☐ Stämmer mycket bra

Stress gör att jag hoppar över måltider eller glömmer bort att äta

☐ Stämmer mycket dåligt

☐ Stämmer ganska dåligt

☐ Stämmer varken bra eller dåligt

☐ Stämmer ganska bra

☐ Stämmer mycket bra

Reklam för onyttig mat får mig att vilja äta onyttigt

☐ Stämmer mycket dåligt

☐ Stämmer ganska dåligt

☐ Stämmer varken bra eller dåligt

☐ Stämmer ganska bra

☐ Stämmer mycket bra

Jag blir ofta bjuden på onyttig mat

☐ Stämmer mycket dåligt

☐ Stämmer ganska dåligt

☐ Stämmer varken bra eller dåligt

☐ Stämmer ganska bra

☐ Stämmer mycket bra

Jag ser ofta onyttig mat i matbutiker och det gör mig sugen på att köpa onyttiga saker

☐ Stämmer mycket dåligt

☐ Stämmer ganska dåligt

☐ Stämmer varken bra eller dåligt

☐ Stämmer ganska bra

☐ Stämmer mycket bra

Jag äter onyttigare när jag äter mat med kompisar än tillsammans med familjen

☐ Stämmer mycket dåligt

☐ Stämmer ganska dåligt

☐ Stämmer varken bra eller dåligt

☐ Stämmer ganska bra

☐ Stämmer mycket bra

Jag äter onyttigare ensam hemma än när jag äter tillsammans med familjen

☐ Stämmer mycket dåligt

☐ Stämmer ganska dåligt

☐ Stämmer varken bra eller dåligt

☐ Stämmer ganska bra

☐ Stämmer mycket bra

Jag äter ofta något onyttigt ”som belöning” när jag gjort något bra

- ☐ Stämmer mycket dåligt
- ☐ Stämmer ganska dåligt
- ☐ Stämmer varken bra eller dåligt
- ☐ Stämmer ganska bra
- ☐ Stämmer mycket bra

Jag äter oftare onyttigt när jag känner mig trött

- ☐ Stämmer mycket dåligt
- ☐ Stämmer ganska dåligt
- ☐ Stämmer varken bra eller dåligt
- ☐ Stämmer ganska bra
- ☐ Stämmer mycket bra

Jag äter oftare onyttigt när jag känner mig ledsen

- ☐ Stämmer mycket dåligt
- ☐ Stämmer ganska dåligt
- ☐ Stämmer varken bra eller dåligt
- ☐ Stämmer ganska bra
- ☐ Stämmer mycket bra

Jag låter ofta bli att äta när jag känner mig ledsen

- ☐ Stämmer mycket dåligt
- ☐ Stämmer ganska dåligt
- ☐ Stämmer varken bra eller dåligt
- ☐ Stämmer ganska bra
- ☐ Stämmer mycket bra

Andra saker får dig att äta onyttigt:____

____
____
____

9. **Nedan följer frågor som handlar om vad som skulle kunna få dig att äta nyttigare?**

Jag skulle äta nyttigare om nyttig mat var godare

- ☐ Stämmer mycket dåligt
- ☐ Stämmer ganska dåligt
- ☐ Stämmer varken bra eller dåligt
- ☐ Stämmer ganska bra
- ☐ Stämmer mycket bra

Jag skulle äta nyttigare om det serverades nyttigare mat hemma

- ☐ Stämmer mycket dåligt
- ☐ Stämmer ganska dåligt
- ☐ Stämmer varken bra eller dåligt
- ☐ Stämmer ganska bra
- ☐ Stämmer mycket bra

Jag skulle äta nyttigare om skolans mat var godare

- ☐ Stämmer mycket dåligt
- ☐ Stämmer ganska dåligt
- ☐ Stämmer varken bra eller dåligt
- ☐ Stämmer ganska bra
- ☐ Stämmer mycket bra

Jag skulle äta nyttigare om det såldes goda nyttiga saker på ett café i skolan

- ☐ Stämmer mycket dåligt
- ☐ Stämmer ganska dåligt
- ☐ Stämmer varken bra eller dåligt
- ☐ Stämmer ganska bra
- ☐ Stämmer mycket bra

Jag skulle äta nyttigare om jag fick mer kunskap om vad som är nyttigt

☐ Stämmer mycket dåligt

☐ Stämmer ganska dåligt

☐ Stämmer varken bra eller dåligt

☐ Stämmer ganska bra

☐ Stämmer mycket bra

Jag skulle äta nyttigare om jag fick mer inspiration om nyttig mat, som smakprov, recept, bilder/filmer eller annan inspiration

☐ Stämmer mycket dåligt

☐ Stämmer ganska dåligt

☐ Stämmer varken bra eller dåligt

☐ Stämmer ganska bra

☐ Stämmer mycket bra

Jag äter redan nyttigt

☐ Stämmer mycket dåligt

☐ Stämmer ganska dåligt

☐ Stämmer varken bra eller dåligt

☐ Stämmer ganska bra

☐ Stämmer mycket bra

Annat som skulle hjälpa dig att äta nyttigare, i så fall vad:________________________________
____
____

10. **Varifrån får du information om mat och hälsa? (fler svar kan väljas)**

sociala medier

- ☐ Snapchat
- ☐ Youtube
- ☐ Instagram
- ☐ Facebook
- ☐ Twitter
- ☐ Kik
- ☐ Hemsidor eller bloggar

TV/streaming

- ☐ Nyheter
- ☐ Dokumentärer
- ☐ Reality, t.ex. Biggest looser

Övrig media

- ☐ Poddar
- ☐ Radio
- ☐ Böcker
- ☐ Tidningar i pappersformat

Personliga informationskällor

- ☐ Lärare, t.ex. i idrott, hemkunskap eller biologi
- ☐ Skolsköterska
- ☐ Annan sjuksköterska/läkare
- ☐ Idrottsklubb (tränare eller annat)
- ☐ Förälder/föräldrar
- ☐ Syskon
- ☐ Kompis/kompisar

____

- ☐ Vet inte
- ☐ Jag bryr mig inte om mat och hälsa
- ☐ Jag letar aldrig efter information om mat och hälsa

Annat, i så fall vad:________________________
____

11. **Inom vilket område blir du inspirerad av mat och hälsa? (fler svar kan väljas)**

- ☐ Hälsa
- ☐ Träning
- ☐ Mat
- ☐ Samhällsinformation, typ nyheter
- ☐ Jag blir inte inspirerad av mat och hälsa

Annat, i så fall vad:____________________________
____

12. **Om du skulle välja EN person från social media (en influencer) som du skulle bli inspirerad av till att äta en speciell mat eller produkt, vem skulle det vara:________________________**

13. Vad är din spontana tanke när det kommer till hälsa och de saker som nämns nedan?
Välj det svarsalternativ som ligger dig närmast (ett kryss), även om det inte stämmer exakt!

Tillsatser

☐ Mycket onyttigt
☐ Ganska onyttigt
☐ Varken nyttigt eller onyttigt
☐ Ganska nyttigt
☐ Mycket nyttigt
☐ Vet inte vad tillsatser är

Kost med lågt innehåll av kolhydrater (t.ex. LCHF)

☐ Mycket onyttigt
☐ Ganska onyttigt
☐ Varken nyttigt eller onyttigt
☐ Ganska nyttigt
☐ Mycket nyttigt
☐ Vet inte vad lågkolhydratkost är

Grönsaker

☐ Mycket onyttigt
☐ Ganska onyttigt
☐ Varken nyttigt eller onyttigt
☐ Ganska nyttigt
☐ Mycket nyttigt
☐ Vet inte vad grönsaker är

Fett

☐ Mycket onyttigt
☐ Ganska onyttigt
☐ Varken nyttigt eller onyttigt
☐ Ganska nyttigt
☐ Mycket nyttigt
☐ Vet inte vad torkad frukt

Protein

☐ Mycket onyttigt

☐ Ganska onyttigt

☐ Varken nyttigt eller onyttigt

☐ Ganska nyttigt

☐ Mycket nyttigt

☐ Vet inte vad protein är

Lättprodukter eller lightprodukter

☐ Mycket onyttigt

☐ Ganska onyttigt

☐ Varken nyttigt eller onyttigt

☐ Ganska nyttigt

☐ Mycket nyttigt

☐ Vet inte vad lätt/lightprodukter är

Torkad frukt

☐ Mycket onyttigt

☐ Ganska onyttigt

☐ Varken nyttigt eller onyttigt

☐ Ganska nyttigt

☐ Mycket nyttigt

☐ Vet inte vad torkad frukt är

Socker

☐ Mycket onyttigt

☐ Ganska onyttigt

☐ Varken nyttigt eller onyttigt

☐ Ganska nyttigt

☐ Mycket nyttigt

☐ Vet inte vad socker är

Nyckelhålet

☐ Mycket onyttigt

☐ Ganska onyttigt

☐ Varken nyttigt eller onyttigt

☐ Ganska nyttigt

☐ Mycket nyttigt

☐ Vet inte vad nyckelhålet innebär

Vegetariskt

☐ Mycket onyttigt

☐ Ganska onyttigt

☐ Varken nyttigt eller onyttigt

☐ Ganska nyttigt

☐ Mycket nyttigt

☐ Vet inte vad vegetariskt är

Surdeg

☐ Mycket onyttigt

☐ Ganska onyttigt

☐ Varken nyttigt eller onyttigt

☐ Ganska nyttigt

☐ Mycket nyttigt

☐ Vet inte vad surdeg är

Fullkorn

☐ Mycket onyttigt

☐ Ganska onyttigt

☐ Varken nyttigt eller onyttigt

☐ Ganska nyttigt

☐ Mycket nyttigt

☐ Vet inte vad fullkorn är

Kolhydrater

☐ Mycket onyttigt

☐ Ganska onyttigt

☐ Varken nyttigt eller onyttigt

☐ Ganska nyttigt

☐ Mycket nyttigt

☐ Vet inte vad kolhydrater är

Lågt glykemiskt index (GI)

☐ Mycket onyttigt

☐ Ganska onyttigt

☐ Varken nyttigt eller onyttigt

☐ Ganska nyttigt

☐ Mycket nyttigt

☐ Vet inte vad GI är

Frukt

☐ Mycket onyttigt

☐ Ganska onyttigt

☐ Varken nyttigt eller onyttigt

☐ Ganska nyttigt

☐ Mycket nyttigt

☐ Vet inte vad frukt är

Naturligt

☐ Mycket onyttigt

☐ Ganska onyttigt

☐ Varken nyttigt eller onyttigt

☐ Ganska nyttigt

☐ Mycket nyttigt

☐ Vet inte vad naturligt är

Fibrer

☐ Mycket onyttigt

☐ Ganska onyttigt

☐ Varken nyttigt eller onyttigt

☐ Ganska nyttigt

☐ Mycket nyttigt

☐ Vet inte vad fibrer är

Salt

☐ Mycket onyttigt

☐ Ganska onyttigt

☐ Varken nyttigt eller onyttigt

☐ Ganska nyttigt

☐ Mycket nyttigt

☐ Vet inte vad salt är

Veganskt

☐ Mycket onyttigt

☐ Ganska onyttigt

☐ Varken nyttigt eller onyttigt

☐ Ganska nyttigt

☐ Mycket nyttigt

☐ Vet inte vad veganskt är

Livsmedelsverkets kostråd

☐ Mycket onyttigt

☐ Ganska onyttigt

☐ Varken nyttigt eller onyttigt

☐ Ganska nyttigt

☐ Mycket nyttigt

☐ Vet inte vad råden innebär

☐ Vet inte vad Livsmedelsverket är eller gör

Gluten

☐ Mycket onyttigt

☐ Ganska onyttigt

☐ Varken nyttigt eller onyttigt

☐ Ganska nyttigt

☐ Mycket nyttigt

☐ Vet inte vad gluten är

Baljväxter (bönor, linser, ärter)

☐ Mycket onyttigt

☐ Ganska onyttigt

☐ Varken nyttigt eller onyttigt

☐ Ganska nyttigt

☐ Mycket nyttigt

☐ Vet inte vad baljväxter är

”Raffinerade/vita” kolhydrater (t.ex. vitt bröd, vitt ris, vit pasta)

☐ Mycket onyttigt

☐ Ganska onyttigt

☐ Varken nyttigt eller onyttigt

☐ Ganska nyttigt

☐ Mycket nyttigt

☐ Vet inte vad raffinerade/vita kolhydrater är

Ekologiskt

☐ Mycket onyttigt

☐ Ganska onyttigt

☐ Varken nyttigt eller onyttigt

☐ Ganska nyttigt

☐ Mycket nyttigt

☐ Vet inte vad ekologiskt är

14. **Vilka livsmedel tror du kan vara fullkorn? (flera svar kan väljas):**

☐ Pasta ☐ Äpple ☐ Pizzadeg ☐ Popcorn ☐ Flingor/müsli ☐ Mjölk ☐ Nötter

☐ Te ☐ Energidryck ☐ Kaffe ☐ Köttbullar ☐ Banan ☐ Bullar ☐ Bröd

☐ Kyckling ☐ Juice ☐ Ris ☐ Bönor ☐ Lax ☐ Torkad frukt ☐ Läsk ☐ Quinoa

☐ Bulgur ☐ Ägg ☐ Yoghurt ☐ Gröt ☐ Linser ☐ Choklad ☐ Ost

Annat, i så fall vad: ________________________________

Utan att du ändrar ditt svar på de två tidigare frågorna vill vi nu berätta vad fullkorn är, och ställa några av frågorna igen. Obs! Ändra inga svar du redan svarat på (ovanför)! Kom ihåg att det inte finns några "rätt och fel" svar och ingen kommer att se hur just du svarat.

15. Ett sädeskorn består av grodd (längst in), mjölkropp (det som blir det vita mjölet) och kli (yttersta lagret). Fullkorn innebär att alla dessa tre delar av ett sädesslag finns kvar i produkten t.ex. ett mjöl. Därför är t.ex. fullkornsbröd eller fullkornspasta ofta lite mörkare i färgen och innehåller mer fibrer.

Hur ofta brukar du välja ett fullkornsalternativ när du äter något av följande:

Bröd

☐ Aldrig

☐ Ibland

☐ Ca hälften av gångerna

☐ Oftast

☐ Alltid

☐ Äter inte bröd

Pasta

☐ Aldrig

☐ Ibland

☐ Ca hälften av gångerna

☐ Oftast

☐ Alltid

☐ Äter inte pasta

Ris

☐ Aldrig

☐ Ibland

☐ Ca hälften av gångerna

☐ Oftast

☐ Alltid

☐ Äter inte ris

Frukostflingor

☐ Aldrig

☐ Ibland

☐ Ca hälften av gångerna

☐ Oftast

☐ Alltid

☐ Äter inte flingor

Gröt

☐ Aldrig

☐ Ibland

☐ Ca hälften av gångerna

☐ Oftast

☐ Alltid

☐ Äter inte gröt

Annat, i så fall vad: ____

☐ Ibland

☐ Ca hälften av gångerna

☐ Oftast

☐ Alltid

16. Hur ofta pratar ni om fullkorn i er familj?

- ☐ Aldrig
- ☐ Ganska sällan
- ☐ Då och då
- ☐ Ganska ofta
- ☐ Väldigt ofta

17. Om du visste att det var mer hälsosamt att välja mat med fullkorn, hur väl stämmer följande alternativ in på dig?

Jag kan tänka mig att äta mer fullkorn om det serverades mer i skolan.

- ☐ Stämmer mycket dåligt
- ☐ Stämmer ganska dåligt
- ☐ Stämmer varken bra eller dåligt
- ☐ Stämmer ganska bra
- ☐ Stämmer mycket bra

Jag kan tänka mig att äta mer fullkorn om det serverades mer hemma

- ☐ Stämmer mycket dåligt
- ☐ Stämmer ganska dåligt
- ☐ Stämmer varken bra eller dåligt
- ☐ Stämmer ganska bra
- ☐ Stämmer mycket bra

Jag kan tänka mig att äta mer fullkorn om det fanns mer alternativ i butiker

- ☐ Stämmer mycket dåligt
- ☐ Stämmer ganska dåligt
- ☐ Stämmer varken bra eller dåligt
- ☐ Stämmer ganska bra
- ☐ Stämmer mycket bra

Jag kan tänka mig att äta mer fullkorn om det fanns mer alternativ på café

☐ Stämmer mycket dåligt

☐ Stämmer ganska dåligt

☐ Stämmer varken bra eller dåligt

☐ Stämmer ganska bra

☐ Stämmer mycket bra

Jag skulle äta mer fullkorn om det smakade godare

☐ Stämmer mycket dåligt

☐ Stämmer ganska dåligt

☐ Stämmer varken bra eller dåligt

☐ Stämmer ganska bra

☐ Stämmer mycket bra

Jag skulle äta mer fullkorn om det såg godare ut

☐ Stämmer mycket dåligt

☐ Stämmer ganska dåligt

☐ Stämmer varken bra eller dåligt

☐ Stämmer ganska bra

☐ Stämmer mycket bra

Jag vill att produkter med fullkorn ska smaka fullkorn.

☐ Stämmer mycket dåligt

☐ Stämmer ganska dåligt

☐ Stämmer varken bra eller dåligt

☐ Stämmer ganska bra

☐ Stämmer mycket bra

Att jag vet om att ett livsmedel innehåller fullkorn lockar mig att äta livsmedlet

☐ Stämmer mycket dåligt

☐ Stämmer ganska dåligt

☐ Stämmer varken bra eller dåligt

☐ Stämmer ganska bra

☐ Stämmer mycket bra

Annat som skulle få mig att äta *mer* fullkorn, i så fall vad: ___________________________________

Annat som skulle få mig att äta *mindre* fullkorn, i så fall vad: ________________________________

Jag vill inte äta mer fullkorn eftersom jag redan äter mycket

☐ Stämmer mycket dåligt

☐ Stämmer ganska dåligt

☐ Stämmer varken bra eller dåligt

☐ Stämmer ganska bra

☐ Stämmer mycket bra

Jag vill inte äta mer fullkorn eftersom jag inte bryr mig om nyttig mat

☐ Stämmer mycket dåligt

☐ Stämmer ganska dåligt

☐ Stämmer varken bra eller dåligt

☐ Stämmer ganska bra

☐ Stämmer mycket bra

Annan orsak till att jag inte vill äta mer fullkorn:____

18. **Hur väl stämmer följande påståenden in på dig när det kommer till att kunna tänka dig att välja fler produkter med fullkorn i?**

Jag kan tänka mig att äta mer bröd med fullkorn än vad jag vanligtvis gör.

☐ Stämmer mycket dåligt

☐ Stämmer ganska dåligt

☐ Stämmer varken bra eller dåligt

☐ Stämmer ganska bra

☐ Stämmer mycket bra

☐ Äter redan mycket bröd med fullkorn

☐ Äter inte bröd

Jag kan tänka mig att äta mer pasta med fullkorn än vad jag vanligtvis gör.

☐ Stämmer mycket dåligt

☐ Stämmer ganska dåligt

☐ Stämmer varken bra eller dåligt

☐ Stämmer ganska bra

☐ Stämmer mycket bra

☐ Äter redan mycket pasta med fullkorn

☐ Äter inte pasta

Jag kan tänka mig att äta mer ris med fullkorn än vad jag vanligtvis gör.

☐ Stämmer mycket dåligt

☐ Stämmer ganska dåligt

☐ Stämmer varken bra eller dåligt

☐ Stämmer ganska bra

☐ Stämmer mycket bra

☐ Äter redan mycket ris med fullkorn

☐ Äter inte pasta

Jag kan tänka mig att äta mer gröt med fullkorn än vad jag vanligtvis gör.

- ☐ Stämmer mycket dåligt
- ☐ Stämmer ganska dåligt
- ☐ Stämmer varken bra eller dåligt
- ☐ Stämmer ganska bra
- ☐ Stämmer mycket bra
- ☐ Äter redan mycket gröt med fullkorn
- ☐ Äter inte gröt

Jag kan tänka mig att äta mer snabbmat typ pizzabotten/hamburgerbröd med fullkorn än vad jag vanligtvis gör.

- ☐ Stämmer mycket dåligt
- ☐ Stämmer ganska dåligt
- ☐ Stämmer varken bra eller dåligt
- ☐ Stämmer ganska bra
- ☐ Stämmer mycket bra
- ☐ Äter redan mycket snabbmat med fullkorn
- ☐ Äter inte snabbmat

Jag kan tänka mig att äta mer flingor/müsli med fullkorn än vad jag vanligtvis gör.

- ☐ Stämmer mycket dåligt
- ☐ Stämmer ganska dåligt
- ☐ Stämmer varken bra eller dåligt
- ☐ Stämmer ganska bra
- ☐ Stämmer mycket bra
- ☐ Äter redan mycket frukostflingor/müsli med fullkorn
- ☐ Äter inte flingor eller müsli

Annat jag kan tänka mig att äta med mer fullkorn i, i så fall vad: ______________________________

FRÅGOR OM SOCKER

19. Vad stämmer bäst med hur ofta du brukar äta/dricka produkter med mycket socker i?

Läsk, saft eller sockrad juice (nektar)

☐ Aldrig eller nästan aldrig

☐ 1-3 gånger per månad

☐ 1-3 gånger per vecka

☐ 4-7 gånger per vecka

☐ Flera gånger per dag

☐ Dricker oftast sockerfri läsk eller saft (med sötningsmedel som aspartam eller stevia)

Oboy eller annan mjölkliknande sockrad dryck

☐ Aldrig eller nästan aldrig

☐ 1-3 gånger per månad

☐ 1-3 gånger per vecka

☐ 4-7 gånger per vecka

☐ Flera gånger per dag

☐ Dricker oftast sockerfria mjölkdrycker (med sötningsmedel som aspartam eller stevia)

Godis (förutom choklad)

☐ Aldrig eller nästan aldrig

☐ 1-3 gånger per månad

☐ 1-3 gånger per vecka

☐ 4-7 gånger per vecka

☐ Flera gånger per dag

☐ Äter oftast sockerfritt godis

Choklad

☐ Aldrig eller nästan aldrig

☐ 1-3 gånger per månad

☐ 1-3 gånger per vecka

☐ 4-7 gånger per vecka

☐ Flera gånger per dag

☐ Äter oftast sockerfri choklad

Bakverk/kakor

☐ Aldrig eller nästan aldrig

☐ 1-3 gånger per månad

☐ 1-3 gånger per vecka

☐ 4-7 gånger per vecka

☐ Flera gånger per dag

☐ Äter oftast sockerfria bakverk/kakor

Glass (under våren och sommaren)

☐ Aldrig eller nästan aldrig

☐ 1-3 gånger per månad

☐ 1-3 gånger per vecka

☐ 4-7 gånger per vecka

☐ Flera gånger per dag

☐ Äter oftast sockerfri glass

Glass (under hösten och vintern)

☐ Aldrig eller nästan aldrig

☐ 1-3 gånger per månad

☐ 1-3 gånger per vecka

☐ 4-7 gånger per vecka

☐ Flera gånger per dag

☐ Äter oftast sockerfri glass

Sylt eller marmelad

☐ Aldrig eller nästan aldrig

☐ 1-3 gånger per månad

☐ 1-3 gånger per vecka

☐ 4-7 gånger per vecka

☐ Flera gånger per dag

☐ Äter oftast sockerfri sylt/marmelad

Flingor/müsli/puffar som är sockrade

- ☐ Aldrig eller nästan aldrig
- ☐ 1-3 gånger per månad
- ☐ 1-3 gånger per vecka
- ☐ 4-7 gånger per vecka
- ☐ Flera gånger per dag
- ☐ Äter oftast osöta frukostflingor

Andra produkter med mycket socker, i så fall vad: ____________________________

- ☐ 1-3 gånger per månad
- ☐ 1-3 gånger per vecka
- ☐ 4-7 gånger per vecka
- ☐ Flera gånger per dag

20. Hur väl stämmer följande påståenden in på dig när det kommer till socker?

Vi pratar ofta om socker och hälsa i vår familj?

- ☐ Stämmer mycket dåligt
- ☐ Stämmer ganska dåligt
- ☐ Stämmer varken bra eller dåligt
- ☐ Stämmer ganska bra
- ☐ Stämmer mycket bra

Jag skulle vilja äta mindre socker för att jag tror att jag blir friskare

- ☐ Stämmer mycket dåligt
- ☐ Stämmer ganska dåligt
- ☐ Stämmer varken bra eller dåligt
- ☐ Stämmer ganska bra
- ☐ Stämmer mycket bra

Jag skulle vilja äta mindre socker för jag tror det är dåligt för tänderna

☐ Stämmer mycket dåligt

☐ Stämmer ganska dåligt

☐ Stämmer varken bra eller dåligt

☐ Stämmer ganska bra

☐ Stämmer mycket bra

Jag skulle vilja äta mindre socker för jag tycker eller tror att jag blir piggare

☐ Stämmer mycket dåligt

☐ Stämmer ganska dåligt

☐ Stämmer varken bra eller dåligt

☐ Stämmer ganska bra

☐ Stämmer mycket bra

Annan orsak till att jag vill äta mindre socker:____

Jag vill inte äta mindre socker eftersom jag redan äter lite eller inget socker

☐ Stämmer mycket dåligt

☐ Stämmer ganska dåligt

☐ Stämmer varken bra eller dåligt

☐ Stämmer ganska bra

☐ Stämmer mycket bra

Jag vill inte äta mindre socker eftersom jag inte tror det spelar någon roll för hälsan

☐ Stämmer mycket dåligt

☐ Stämmer ganska dåligt

☐ Stämmer varken bra eller dåligt

☐ Stämmer ganska bra

☐ Stämmer mycket bra

Jag vill inte äta mindre socker eftersom jag inte bryr mig om mat och hälsa

- ☐ Stämmer mycket dåligt
- ☐ Stämmer ganska dåligt
- ☐ Stämmer varken bra eller dåligt
- ☐ Stämmer ganska bra
- ☐ Stämmer mycket bra

Jag vill inte äta mindre socker eftersom det skulle göra mig tröttare

- ☐ Stämmer mycket dåligt
- ☐ Stämmer ganska dåligt
- ☐ Stämmer varken bra eller dåligt
- ☐ Stämmer ganska bra
- ☐ Stämmer mycket bra
- ☐ Äter redan lite/inget

Annan orsak till att jag inte vill äta mindre socker:________________________

21. Hur väl stämmer följande påståenden in på dig när det kommer till vad som skulle få dig att äta mindre socker?

Jag skulle äta mindre socker om det serverades mindre hemma

- ☐ Stämmer mycket dåligt
- ☐ Stämmer ganska dåligt
- ☐ Stämmer varken bra eller dåligt
- ☐ Stämmer ganska bra
- ☐ Stämmer mycket bra
- ☐ Äter redan lite/inget

Jag skulle äta mindre socker om det fanns goda sockerfria alternativ att köpa i skolan

☐ Stämmer mycket dåligt

☐ Stämmer ganska dåligt

☐ Stämmer varken bra eller dåligt

☐ Stämmer ganska bra

☐ Stämmer mycket bra

☐ Äter redan lite/inget

Jag skulle äta mindre socker om det fanns goda sockerfria alternativ i butiker

☐ Stämmer mycket dåligt

☐ Stämmer ganska dåligt

☐ Stämmer varken bra eller dåligt

☐ Stämmer ganska bra

☐ Stämmer mycket bra

☐ Äter redan lite/inget

Jag skulle äta mindre socker om det fanns goda sockerfria alternativ på caféer

☐ Stämmer mycket dåligt

☐ Stämmer ganska dåligt

☐ Stämmer varken bra eller dåligt

☐ Stämmer ganska bra

☐ Stämmer mycket bra

☐ Äter redan lite/inget

Annat:
Jag skulle äta mindre socker om:________________________________

22. **Äter du något av följande söta livsmedel?**

Kokossocker

- ☐ Aldrig
- ☐ 1-3 gånger per månad
- ☐ 1-3 gånger per vecka
- ☐ 4-7 gånger per vecka
- ☐ Flera gånger per dag
- ☐ Vet inte vad kokossocker är

Kokossirap

- ☐ Aldrig
- ☐ 1-3 gånger per månad
- ☐ 1-3 gånger per vecka
- ☐ 4-7 gånger per vecka
- ☐ Flera gånger per dag
- ☐ Vet inte vad kokossirap är

Rårörsocker

- ☐ Aldrig
- ☐ 1-3 gånger per månad
- ☐ 1-3 gånger per vecka
- ☐ 4-7 gånger per vecka
- ☐ Flera gånger per dag
- ☐ Vet inte vad rårörsocker är

Torkad frukt

- ☐ Aldrig
- ☐ 1-3 gånger per månad
- ☐ 1-3 gånger per vecka
- ☐ 4-7 gånger per vecka
- ☐ Flera gånger per dag
- ☐ Vet inte vad torkad frukt är

Honung

- ☐ Aldrig
- ☐ 1-3 gånger per månad
- ☐ 1-3 gånger per vecka
- ☐ 4-7 gånger per vecka
- ☐ Flera gånger per dag
- ☐ Vet inte vad honung är

Agavesirap

- ☐ Aldrig
- ☐ 1-3 gånger per månad
- ☐ 1-3 gånger per vecka
- ☐ 4-7 gånger per vecka
- ☐ Flera gånger per dag
- ☐ Vet inte vad agavesirap är

Lönnsirap

- ☐ Aldrig
- ☐ 1-3 gånger per månad
- ☐ 1-3 gånger per vecka
- ☐ 4-7 gånger per vecka
- ☐ Flera gånger per dag
- ☐ Vet inte vad lönnsirap är

Fruktjuice

- ☐ Aldrig
- ☐ 1-3 gånger per månad
- ☐ 1-3 gånger per vecka
- ☐ 4-7 gånger per vecka
- ☐ Flera gånger per dag
- ☐ Vet inte vad fruktjuice är

Sötningsmedel från naturliga källor, typ stevia

☐ Aldrig

☐ 1-3 gånger per månad

☐ 1-3 gånger per vecka

☐ 4-7 gånger per vecka

☐ Flera gånger per dag

☐ Vet inte vad naturliga sötningsmedel är

Artificiella sötningsmedel, typ aspartam

☐ Aldrig

☐ 1-3 gånger per månad

☐ 1-3 gånger per vecka

☐ 4-7 gånger per vecka

☐ Flera gånger per dag

☐ Vet inte vad artificiella sötningsmedel är

Dadelsocker/pulver

☐ Aldrig

☐ 1-3 gånger per månad

☐ 1-3 gånger per vecka

☐ 4-7 gånger per vecka

☐ Flera gånger per dag

☐ Vet inte vad dadelsocker/pulver är

Dadelsirap

☐ Aldrig

☐ 1-3 gånger per månad

☐ 1-3 gånger per vecka

☐ 4-7 gånger per vecka

☐ Flera gånger per dag

☐ Vet inte vad dadelsirap är

Annat som du äter för att göra maten sötare, i så fall vad: ____________________

☐ 1-3 gånger per månad

☐ 1-3 gånger per vecka

☐ 4-7 gånger per vecka

☐ Flera gånger per dag

23. Vad är din uppfattning om följande söta produkter <u>när det kommer till hälsa/nyttighet</u>?

Kokossocker

☐ Mycket onyttigt

☐ Ganska onyttigt

☐ Varken nyttigt eller onyttigt

☐ Ganska nyttigt

☐ Mycket nyttigt

☐ Vet inte vad kokosocker är

Kokossirap

☐ Mycket onyttigt

☐ Ganska onyttigt

☐ Varken nyttigt eller onyttigt

☐ Ganska nyttigt

☐ Mycket nyttigt

☐ Vet inte vad kokossirap är

Rårörsocker

☐ Mycket onyttigt

☐ Ganska onyttigt

☐ Varken nyttigt eller onyttigt

☐ Ganska nyttigt

☐ Mycket nyttigt

☐ Vet inte vad rårörsocker är

Torkad frukt

☐ Mycket onyttigt

☐ Ganska onyttigt

☐ Varken nyttigt eller onyttigt

☐ Ganska nyttigt

☐ Mycket nyttigt

☐ Vet inte vad torkad frukt är

Honung

☐ Mycket onyttigt

☐ Ganska onyttigt

☐ Varken nyttigt eller onyttigt

☐ Ganska nyttigt

☐ Mycket nyttigt

☐ Vet inte vad fast honung är

Agavesirap

☐ Mycket onyttigt

☐ Ganska onyttigt

☐ Varken nyttigt eller onyttigt

☐ Ganska nyttigt

☐ Mycket nyttigt

☐ Vet inte vad agavesirap är

Lönnsirap

☐ Mycket onyttigt

☐ Ganska onyttigt

☐ Varken nyttigt eller onyttigt

☐ Ganska nyttigt

☐ Mycket nyttigt

☐ Vet inte vad lönnsirap är

Fruktjuice

- ☐ Mycket onyttigt
- ☐ Ganska onyttigt
- ☐ Varken nyttigt eller onyttigt
- ☐ Ganska nyttigt
- ☐ Mycket nyttigt
- ☐ Vet inte vad fruktjuice är

Sötningsmedel från naturliga källor, typ stevia

- ☐ Mycket onyttigt
- ☐ Ganska onyttigt
- ☐ Varken nyttigt eller onyttigt
- ☐ Ganska nyttigt
- ☐ Mycket nyttigt
- ☐ Vet inte vad naturliga sötningsmedel är

Artificiella sötningsmedel, typ aspartam

- ☐ Mycket onyttigt
- ☐ Ganska onyttigt
- ☐ Varken nyttigt eller onyttigt
- ☐ Ganska nyttigt
- ☐ Mycket nyttigt
- ☐ Vet inte vad artificiella sötningsmedel är

Dadelsocker/pulver

- ☐ Mycket onyttigt
- ☐ Ganska onyttigt
- ☐ Varken nyttigt eller onyttigt
- ☐ Ganska nyttigt
- ☐ Mycket nyttigt
- ☐ Vet inte vad dadelsocker/pulver är

Dadelsirap

☐ Mycket onyttigt

☐ Ganska onyttigt

☐ Varken nyttigt eller onyttigt

☐ Ganska nyttigt

☐ Mycket nyttigt

☐ Vet inte vad dadelsirap är

Strösocker

☐ Mycket onyttigt

☐ Ganska onyttigt

☐ Varken nyttigt eller onyttigt

☐ Ganska nyttigt

☐ Mycket nyttigt

☐ Vet inte vad strösocker är

Pärlsocker

☐ Mycket onyttigt

☐ Ganska onyttigt

☐ Varken nyttigt eller onyttigt

☐ Ganska nyttigt

☐ Mycket nyttigt

☐ Vet inte vad pärlsocker är

Florsocker

☐ Mycket onyttigt

☐ Ganska onyttigt

☐ Varken nyttigt eller onyttigt

☐ Ganska nyttigt

☐ Mycket nyttigt

☐ Vet inte vad florsocker är

Farinsocker

☐ Mycket onyttigt

☐ Ganska onyttigt

☐ Varken nyttigt eller onyttigt

☐ Ganska nyttigt

☐ Mycket nyttigt

☐ Vet inte vad farinsocker är

Annat som du använder till att söta din mat som du tror är hälsosammare än vanligt vitt socker, i så fall vad: ____________________

Till sist vill vi ställa en fråga om Nyckelhålet . Det är en märkning som kan vara svart eller grön som du kan hitta på olika typer av produkter i affären.

24. Vad tror du att Nyckelhålet betyder (flera svar kan väljas):

☐ Mer fibrer ☐ Mindre fibrer ☐ Mer fullkorn ☐ Mindre fullkorn ☐ Från Sverige

☐ Mer frukt och grönsaker ☐ Mindre frukt och grönsaker ☐ Mer socker ☐ Mindre socker

☐ Mer fett ☐ Mindre fett ☐ Mer tillsatser ☐ Mindre tillsatser ☐ Mer protein

☐ Mindre protein ☐ Ekologisk ☐ Mer salt ☐ Mindre salt ☐ Mer kolhydrater

☐ Mindre kolhydrater ☐ Mer kött ☐ Mindre kött ☐ Lightprodukter ☐ Närodlad

Annat, i så fall vad: __________________________________

☐ **Jag har använt mig av Google translate.**

Dessa ord behövde jag söka förklaring till (lista alla ord nedan):

CHALMERS GÖTEBORGS UNIVERSITET NOBEL PRIZE MUSEUM
